# Naxitamab plus stepped-up dosing of granulocyte–macrophage colony-stimulating factor for primary refractory high-risk neuroblastoma: results of a phase I/II trial

**DOI:** 10.1186/s13045-025-01770-7

**Published:** 2025-11-26

**Authors:** Brian H. Kushner, Shakeel Modak, Audrey Mauguen, Ellen M. Basu, Stephen S. Roberts, Nai-Kong V. Cheung

**Affiliations:** 1 Department of Pediatrics, Memorial Sloan Kettering Cancer Center, 1275 York Avenue, New York, NY 10065 USA; 2 Department of Biostatics and Epidemiology, Memorial Sloan Kettering Cancer Center, 1275 York Avenue, New York, NY 10065 USA; 3 Present Address: Department of Pediatrics, Oregon Health & Science University, Knight Cancer Institute, Portland, OR 97239 USA

**Keywords:** Neuroblastoma, Naxitamab, Immunotherapy, Anti-G_D2_ antibody, GM-CSF

## Abstract

**Background:**

With high-risk neuroblastoma, post-induction metastases in bone marrow (BM)/bones confers a poor prognosis but can respond to anti-G_D2_ antibody, such as naxitamab. We report results of a phase I/II trial.

**Methods:**

Cycles included GM-CSF and naxitamab infused (30-to-90 min) on days + 1, + 3, + 5. Naxitamab was dose-escalated in the trial’s phase I portion and administered at 9 mg/kg/cycle (i.e., ~ 270 mg/m^2^/cycle)—the recommended phase II dosage (RP2D)—in the phase II expansion. Cycles were monthly × 5 after a major response, i.e., complete (CR) or partial response.

**Results:**

Among 32 subjects, CR was noted in 24 (75%), including 12 by international criteria and 12 based on MIBG-avid sites with negative PET scans. BM CR was achieved in 22/23 with BM metastases. Of 29 patients with abnormal ^123^I-MIBG scans, major responses occurred in 14/20 with Curie scores (CS) 10–25 and in 7/9 with CS 1–9. Of 9 patients previously treated with other anti-G_D2_ antibodies, 5 became event-free survivors. Post-protocol, 18 patients received anti-neuroblastoma vaccine. Five-year progression-free/overall survival rates were 38%/64%. Baseline CS, prior 2nd-line therapy, and prior anti-G_D2_ antibody did not significantly impact survival.

**Conclusions:**

Naxitamb + GM-CSF is an attractive option for primary refractory osteomedullary disease, including in patients with a high disease burden.

**Clinical trials registration:**

Clinicaltrials.gov NCT01757626.

## Background

With high-risk neuroblastoma (HR-NB), ~ 50% of patients have post-induction disease detected by bone marrow (BM) studies or ^123^I-metaiodobenzylguanidine (MIBG) scan [1–3]. This primary refractory disease carries a poor prognosis [4]. Yet chemoresistant NB in BM is an attractive target for immunotherapy mediated by monoclonal antibody (mAb) because the BM compartment is immersed in blood. This optimizes accessibility of residual NB to mAb and effector cells (neutrophils and macrophages), thus bypassing a major limitation of immunotherapy, namely, poor trafficking into bulky soft tissue tumor. Indeed, early [5] and more recent studies [6] of anti-G_D2_ mAbs showed promise against disease in BM, but not against NB in soft tissue. Efficacy against large metastases in cortical bone is uncertain.

Cooperative group studies confirmed efficacy in HR-NB patients of anti-G_D2_ mAbs dinutuximab [7] and dinutuximab beta [8]. These chimeric mAbs were administered, respectively, with granulocyte–macrophage colony-stimulating factor (GM-CSF) plus interleukin-2 (IL-2) [7], with IL-2 only [8], and without GM-CSF or IL-2 [8]. The murine anti-G_D2_ IgG3 mAb 3F8 plus GM-CSF proved effective for consolidating 1 st or ≥ 2nd remission of HR-NB [9, 10] and can ablate primary refractory disease in BM [11]. Host reactions against anti-G_D2_ mAbs can induce anti-drug antibodies that might limit efficacy by reducing serum levels. To avoid sensitization, we constructed naxitamab (previously called humanized-3F8) which is an IgG1 subclass humanized form of 3F8. [12]

Preclinical studies revealed differences between naxitamab and other anti-G_D2_ mAbs [12]. Features that made naxitamab attractive for clinical use included superior antibody-dependent cellular cytotoxicity (ADCC) and reduced but active complement-dependent cytotoxicity (CDC). Less CDC might reduce the pain toxicity which is attributed to complement activation [13] and can complicate infusions of anti-G_D2_ mAbs. We, therefore, hypothesized that naxitamab in high dosages would be tolerable and would achieve major anti-NB effects given the dose–response relation between anti-G_D2_ mAbs and ADCC in vitro. [14] Indeed, in the phase I trial [15], no maximum-tolerated dosage was identified and the recommended phase II dosage (RP2D) of 9 mg/kg/cycle (i.e., ~ 270 mg/m^2^/cycle), which is > 2.5 × the standard for other anti-G_D2_ mAbs [7–9], was based in plateauing serum levels. Favorable efficacy and safety findings in an international phase II trial [16] led to approval by the Food and Drug Administration for a specific subpopulation of relapsed/refractory HR-NB with residual bone or BM disease in patients without progression after prior therapy.

Several observations support combining GM-CSF with anti-G_D2_ mAbs. This cytokine is well tolerated clinically and augments granulocyte-mediated ADCC of NB, where killing correlates with effector:target ratios [14]. GM-CSF use has varied in HR-NB clinical trials. It has not been reported with dinutuximab beta. With dinutuximab, GM-CSF dosage has been 250 µg/m^2^/day, with intravenous or subcutaneous administration [7, 17]. In contrast, with 3F8, GM-CSF was administered intravenously in initial studies and subcutaneously in later studies, and always with a step-up from priming dosages of 250 µg/m^2^/day to 500 µg/m^2^/day in the week of 3F8 infusions [9–11]. This doubling of GM-CSF dosage was adopted to exploit the GM-CSF dose–response effect on ADCC [14]. The subcutaneous route and the step-up in dosage resulted in greater activation of granulocytes which was an independent prognostic factor for superior progression-free survival (PFS) compared to intravenously-administered GM-CSF or no GM-CSF [9–11, 18]. A randomized trial would be required to assess if the higher GM-CSF dosage is more effective than the standard dosage.

We now report results of a phase I/II trial of naxitamab + GM-CSF in patients who have primary refractory NB in bones and/or BM.

### Patients and methods

This report covers all HR-NB patients enrolled on the Memorial Sloan Kettering Cancer Center (MSK) protocol 12–230 (Clinicaltrials.gov NCT01757626) with evaluable chemo-resistant disease in bones and/or BM, but not in soft tissue and no prior progressive disease (PD). This trial opened as a phase I study, followed by a phase II expansion (using the RP2D [15]) which included 3 groups: patients with primary refractory disease in BM (Group 1) (subjects of the current report including a subset of 8 patients not analyzed in the phase I report [15]); patients in 2nd or later CR (Group 2), and patients with persistence of disease despite salvage therapy for relapse (Group 3). Results with Groups 2 and 3 will be presented in separate reports. The primary objectives for Group 1 were to assess the activity of naxitamab + GM-CSF in patients who have primary refractory disease in BM by measuring response and by calculating PFS. Eligibility criteria included major organ dysfunction grade ≤ 2 by Common Toxicity Criteria for Adverse Events Version 4.0; and no systemic therapy for ≥ 3 weeks before enrollment. The study was approved and informed written consents were obtained according to MSK Institutional Review Board rules.

### Protocol treatment

Each cycle included subcutaneously-administered priming doses of yeast-derived recombinant GM-CSF (sargramostim; Berlex Laboratories, Seattle, WA) at 250 µg/m^2^/day on days −4 to 0 (Wednesday through Sunday), followed by a step-up to 500 µg/m^2^/day on days + 1 to + 5 (Monday through Friday). GM-CSF was withheld if the absolute neutrophil count was > 20,000/µl. Naxitamab was infused intravenously over 30–90 min on days + 1, + 3, and + 5 (Monday-Wednesday-Friday, i.e., only 3 doses/cycle). Naxitamab was dose-escalated in the phase I portion [15], yielding a RP2D of 3 mg/kg/infusion (9 mg/kg/cycle—i.e., ~ 270 mg/m^2^/cycle) for the phase II expansion. Premedication included opiates and antihistamines. [19]

Treatment was monthly and could continue without a specified maximum number of cycles until PD, unacceptable toxicity, or 5 cycles after a major response, i.e., complete response (CR) or partial response (PR). Human anti-human antibody (HAHA) was measured (as previously described [15]) after every cycle. If HAHA became positive, cycles were deferred until it became negative. As in the predecessor study using 3F8 (MSK protocol 13–260, NCT02100930), if anti-drug positivity precluded timely treatments with naxitamab + GM-CSF, patients were allowed to receive low-dose maintenance regimens such as irinotecan-temozolomide [20]. They could also receive rituximab to hasten suppression of HAHA via effects on B cells. Patients resumed treatment with naxitamab + GM-CSF when HAHA became negative.

This trial allowed radiotherapy, administered to the primary site with an option to irradiate metastatic sites, in accordance with standard practice for HR-NB. [21, 22]

### Treatment response assessment

Extent-of-disease evaluations (performed as described [23]) were required post-cycles 2 and 4 and then every 3 months and included ^123^I-MIBG scan with SPECT imaging (or ^18^F-fluorodeoxyglucose positron emission tomography [PET]) and BM histology. Response was defined by the International Neuroblastoma Response Criteria (INRC) [24] with MIBG findings quantitated by Curie score (CS): CR, no evidence of NB; PR, no new lesions, and > 50% reduction in MIBG absolute bone score (relative Curie bone score ≥ 0.1 to ≤ 0.5) or ≥ 50% reduction in number of PET–avid bone lesions, and BM histology showing CR or minimal disease (> 0 to < 5% tumor infiltration); minor response, no new lesions, CR or PR in MIBG or PET scan but stable disease (SD) in BM, or CR in BM but SD in MIBG or PET scan; SD, neither sufficient shrinkage for PR nor sufficient increase for progressive disease (PD) of non-primary lesions; or PD, new site of disease or > 20% increase in an existing lesion.

CR was also scored for MIBG-positivity, BM CR (based on 4 sites [23], and therefore more extensive than the standard 2 sites of INRC [24]), and negative PET. The utility of PET for identifying patients with residual MIBG-positivity but no active HR-NB has been reported in a small series of 8 HR-NB patients. [25]

### Biostatistics

This is the final analysis of the primary refractory cohort of the study, which is closed for accrual. Because eligibility criteria for treatment in the phase I part of the study were the same as for the phase II portion, this report covers patients included in both phases. The trial was designed as a one-stage trial with two correlated co-primary endpoints (response and PFS). A total of 47 patients was needed to test the null hypothesis that the rate of CR = 67% and 2-year PFS = 48% with 10% type I error and > 90% power. A total of ≥ 40 patients in CR or ≥ 28 patients progression-free at 2 years were needed for the regimen to be promising. As the accrual was terminated early when the subsequent international trial [16] opened, these pre-determined decision rules cannot be applied and estimates of response rate and PFS are reported. PFS was defined as time from registration to PD or death. Patients alive without PD were censored at date of last follow-up. Overall survival (OS) was defined as time from registration to death. Patients alive were censored at date of last follow-up. Survival rates were estimated using Kaplan–Meier estimator. The association of clinical factors and survival, as unplanned exploratory analysis, was assessed using Cox proportional hazards models. The clinical factors were naxitamab dosage (RP2D versus lower than RP2D), age at diagnosis, time from diagnosis, baseline CS, prior 2nd-line therapy, and prior anti-G_D2_ mAb. Estimates are given with two-sided 95% confidence intervals (95%CI), using the exact methods for binary endpoints and based on the log-survival for survival endpoints.

## Results

### Patient characteristics

At the start of protocol therapy (4/23/14-8/6/19), the 32 subjects were 4.6-to-35.4 (median 7.6) months post-diagnosis and 2.4–10.9 (median 4.7) years old (Table 1). Ages at initial diagnosis were 1.7-to-10.5 (median 3.8) years. Included were 6 patients who received lower than the RP2D, ranging from 4.8 mg/kg/cycle to 8.4 mg/kg/cycle, compared to the RP2D of 9 mg/kg/cycle received by 26 patients. Three patients had BM involvement but negative MIBG scans. Twenty-five patients had CS 1–2 or ≥ 3 which, respectively, were prognostically unfavorable post-induction in patients who subsequently underwent tandem MAT [26] or single MAT [27]. Extensive involvement (i.e., a high disease burden) was noted in 20 patients (CS 10–25).

**Table 1:** Characteristics of the 32 study patients

Males:females (ratio)	18:14 (1.3)
*MYCN* amplification	3(9%)
Age at initial diagnosis: median (range)	3.8 (1.7–10.5) years
Age at 1 st dose of naxitamab: median (range)	4.7 (2.4–10.9) years
Time from diagnosis to naxitamab: median (range)	7.6 (4.6–35.4) months
Curie score at enrollment median (range)	13 (0.25)
Curie 0	3^a^
Curie 1 or 2	4
Curie 3–9	5
Curie 10–19	14
Curie 20–25	6
Naxitamab dosage	
Suboptimal/< RP2D	6 (19%)
Optimal/RP2D	26 (81%)
Prior treatment	
Induction chemotherapy	
COG	18 (56%)
MSK	12 (38%)
Other	2 (6%)
Second-line chemotherapy^b^	23 (72%)
^131^I-MIBG therapy	10 (31%)
Anti-G_D2_ monoclonal antibody^b^	9 (28%)
Autologous stem-cell transplantation	1 (3%)

RP2D recommended phase II dosage

^a^All had metastatic involvement of bone marrow

^b^Used for refractory disease

Prior treatment included high-risk induction chemotherapy in all patients (ANBL0532 [n = 18] [21], MSK regimen [n = 12] [28], other [n = 2]); 2nd-line therapy in 23 (72%) patients; ^131^I-MIBG therapy in 12 (37%) patients, and anti-G_D2_ mAb (dinutuximab or m3F8) in 9 (28%) patients (administered with irinotecan-temozolomide—i.e., chemoimmunotherapy [29]—in 4) (Table 1). Only 1 patient had received myeloablative therapy (MAT) with stem-cell transplantation.

### Responses

Among the 32 study patients, best overall responses were CR in 24 (75%, 95%CI: 57–89%), including 12 by INRC [24] and 12 who had MIBG-positivity, BM CR, and negative PET scans [23]; PR in 4 (12%, 95%CI: 4–29%) patients; SD in 3 (9%, 94%CI: 2–25%) patients; and PD in 1 (3%, 95%CI: 0–16%) patient (Table 2). Responses among the 6 phase I patients who received lower than the RP2D were 4 CR, 1 PR, and 1 PD. Major response (CR or PR) was documented post-cycle 2 in 19 patients, post-cycle 3 in 3 patients, post-cycle 4 in 2 patients, and post-cycles 5 and 6 in 1 patient each. Median time to major response was 1.5 months (i.e., post-cycle 2), with range 1.5–5 months.

**Table 2:** Responses^a^

Sites of disease at enrollment	#patients	CR		PR	SD	PD	RT/1 or 2
		INRC	PET (-)				bony site(s)
BM only	3	3	n/a	0	0	0	0
MIBG only							
Curie 1–9	3	1	1	0	0	1	0
Curie 10–24	6	1	2	1	2	0	2
MIBG & BM							
Curie 1–9	6	3	1	1	1^b^	0	4
Curie 11–25	14	4	8	2	0	0	6
Total	32	12	12	4	3	1	12
Response rate (95% CI)		37% (21–56%)	37% (21–56%)	12% (4–29%)	9% (2–25%)	3% (0–16%)	

BM bone marrow, CI confidence interval, CR complete remission, INRC International Neuroblastoma Response Criteria, n/a not applicable, PET positron emission tomography, PD progressive disease, PR partial remission, SD stable disease

^a^No patient had minor response

^b^Came off study after 1 cycle because of HAHA-positivity

BM showed NB at enrollment in 23/32 patients (Table 2). One patient came off study after 1 cycle because of HAHA-positivity and BM was still positive; BM CR was achieved in all the other 22 patients (22/23 = 96%, 95%CI: 78–100%), including 21 after only 2 cycles.

**Fig. 1:**
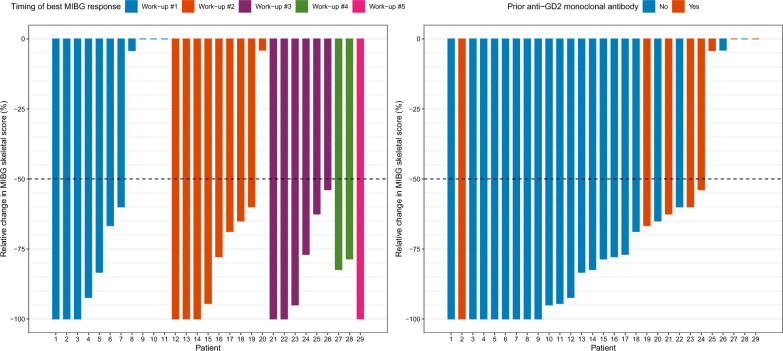
Waterfall plot showing reduction in Curie score in each individual patient, notably including major responses in patients with a high disease burden

MIBG scans showed osteomedullary involvement in 29/32 patients (Table 2); Fig. 1 depicts individual responses. Of 20 patients with extensive MIBG-positivity (CS 10–25), 14 achieved a major response (CR or PR, 14/20 = 70%, 95%CI: 46–88%) including 11 after only 2 cycles. Among 9 patients with less extensive MIBG-positivity (CS 1–9), 7 achieved a major response (7/9 = 78%, 95%CI: 40–97%), including 6 after only 2 cycles.

Nine patients were enrolled post-treatment with other anti-G_D2_ mAbs. One had received both dinutuximab/irinotecan-temozolomide and 3F8 and, with naxitamab + GM-CSF, achieved CR (BM disease alone) followed by vaccine but relapsed at 38 months. Of the 3 other patients (CS 13, 13, and 24 at enrollment) previously treated with dinutuximab/irinotecan-temozolomide, 2 became long-term event-free survivors (54 + and 63 + months post-enrollment). Three of 5 patients previously treated with 3F8 achieved long-term event-free survival (> 6 years post-enrollment), including 1 enrolled with only BM involvement and 2 enrolled with CS 8 and 18.

Consolidative radiotherapy to the primary site was administered post-enrollment in 25 patients, including after cycle 1 (n = 16), cycle 2 (n = 4), cycle 3 (n = 3), cycle 4 (n = 1), and cycle 6 (n = 1). In addition, 3 patients received this treatment before enrollment and 4 patients did not receive it. Radiotherapy was also administered to 1 bony site (8 patients) or 2 bony sites (4 patients); all 12 of these patients remained evaluable for response by INRC [24] because of BM involvement in 10 and Curie scores 7 and 20, respectively, in the other 2 who did not have BM metastases. Seven later developed PD.

Sites of relapse were a single bony site, with 3/6 becoming long-term survivors; CNS alone, with 2/3 becoming long-term survivors; soft tissue alone, with 0/3 survivors; and widespread, with 2/7 becoming long-term survivors. These results came with salvage treatments based on the characteristics of each patient. Among these 19 patients, time to PD from enrollment was 3.5-to-38 (median 11) months and time from a major response (CR or PR) to PD was 2.5-to-37 (median 10) months.

### Survival

Of 22 patients who completed protocol therapy without PD, 21 received subsequent therapy. Their post-protocol treatments included an anti-NB vaccine [30] alone (17 patients); vaccine followed by DFMO [31] (2 patients); dinutuximab + GM-CSF followed by vaccine (the 1 patient who came off study after 1 cycle because of HAHA-positivity); and isotretinoin alone (1 patient). Thirteen of the 32 study patients remained progression-free post-enrollment, including 12 at 4.5 + -to-8.6 + years and 1 who died of pulmonary toxicity unrelated to mAbs at 2.1 years. Eight of these 13 patients had a response of CR based on MIBG-positive sites with negative PET.

**Fig. 2:**
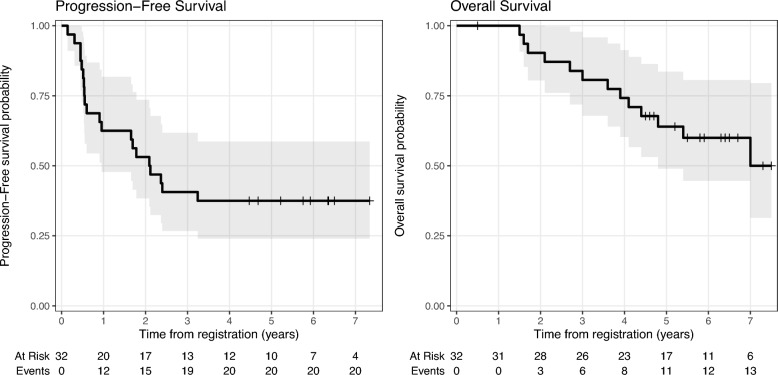
PFS and OS for the whole cohort, with 5-year PFS/OS rates of 38/64%

Figure 2 shows PFS and OS. PFS for the whole cohort was 62% (95%CI: 48%−82%) at 1 year, 53% (95%CI: 38–74%) at 2 years, and 38% (95%CI: 24–59%) at 5 years. OS was 100% at 1 year, 90% (95%CI: 80–100%) at 2 years, and 64% (95%CI: 49–84%) at 5 years. None of the studied clinical factors had a significant impact on PFS or OS (Table 3). For time from diagnosis and CS, non-linear relations were also assessed and no significant associations with PFS or OS were found. Only 3 patients had *MYCN* amplification and, therefore, the association with survival was not assessed. Among them, 2 had PD and none died.

**Table 3:** Prognostic impact of the clinical factors on PFS and OS (Cox regression models; n = 32 for each model)

Characteristic	PFS model				OS model			
	2-year PFS rate (95% CI)	HR	95% CI	p-value	2-year OS rate	HR	95% CI	p-value
**Dosage**								
RP2D	54% (38%, 77%)	–	–		92% (82%, 100%)	–	–	
Lower than RP2D	50% (22%, 100%)	0.75	0.22, 2.55	0.64	83% (58%, 100%)	0.44	0.08, 2.35	0.34
**Age (years)**		1.07	0.88, 1.30	0.51		1.03	0.80, 1.33	0.82
**Time from diagnosis (months)**		1.03	0.93, 1.14	0.62		1.04	0.94, 1.15	0.40
**Baseline Curie score**		1.02	0.96, 1.08	0.46		1.03	0.95, 1.11	0.50
**2nd-line therapy**								
No	33% (13%, 84%)	–	–		78% (55%, 100%)	–	–	
Yes	61% (44%, 84%)	0.57	0.22, 1.44	0.23	95% (87%, 100%)	0.60	0.16, 2.21	0.44
**Prior anti-GD2 monoclonal antibody**^a^								
No	48% (31%, 73%)	–	–		91% (80%, 100%)	–	–	
Yes	67% (42%, 100%)	0.52	0.17, 1.56	0.24	89% (71%, 100%)	0.45	0.14, 1.42	0.17

HR Hazard Ratio, CI Confidence Interval, RP2D recommended phase II dosage

^a^m3F8 or dinutuximab

**Fig. 3:**
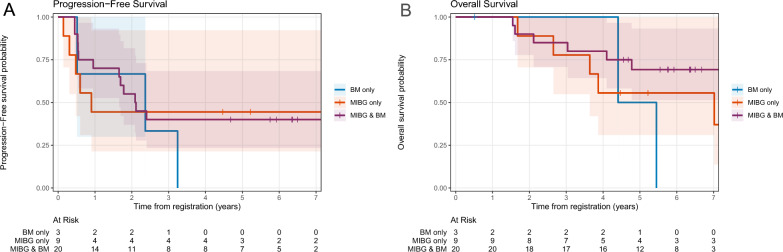
PFS (**A**) and OS (**B**) for subsets of patients. The 5-year PFS/OS rates were 40/69% for the largest group, i.e., patients with disease evident by MIBG scan and BM studies

Figure 3 shows PFS and OS of patients with different findings at enrollment. The largest group was patients with disease documented by MIBG scan and BM histology. For these 22 patients, PFS was 55% (95%CI: 37–82%) at 2 years and 40% (95%CI: 23–68%) at 5 years. Their OS was 90% (95%CI: 78–100%) at 2 years and 69% (95%CI: 51–93%) at 5 years.

### HAHA and toxicity

Patients received 1-to-15 (median 7) cycles of naxitamab + GM-CSF, total 224 cycles. HAHA that interfered with early therapy developed in 3 (9%) patients, post-cycles 1 and 2, including 1 who had prior treatment with an anti-G_D2_ mAb (3F8). Two became HAHA-negative after 2 cycles of rituximab and irinotecan-temozolomide [20] and both then resumed naxitamab + GM-CSF, while 1 came off study (see above) [20]. Only 1 other patient became HAHA-positive, post-cycle 9, and PD was documented then as well. This patient had prior treatment with 3F8.

Side effects were manageable, allowing outpatient treatment. No patient came off study because of toxicity such as anaphylaxis. Common side effects were, as expected with naxitamab [15, 19] and other anti-G_D2_ mAbs [6, 17, 22], grade 1–3 pain, hypo/hypertension, urticaria, fever, and bronchospasm. One patient had to be admitted because of side effects (hypertension). Grade 1–2 Adie’s pupil (mydriasis, photophobia, abnormal accommodation) was documented in 2 patients. Two cycles were shortened due to respiratory symptoms and 1 cycle was shortened due to bradycardia. One patient developed allergy to GM-CSF. The stepped-up dosing of GM-CSF had no associated toxicity, hematological or otherwise. No patient had capillary leak syndrome.

## Discussion

In this study, naxitamab + GM-CSF showed anti-NB activity in HR-NB patients with primary refractory NB in osteomedullary sites. Major responses (CR or PR) were seen in patients with a high disease burden (CS 10–25). Acute toxicities were manageable, allowing outpatient treatment. No adverse sequelae occurred with the stepped-up dosage of GM-CSF. HAHA-positivity was rare. The experience can provide insights into which HR-NB patients might benefit from anti-G_D2_ mAbs + GM-CSF. Given incomplete response rates of ~ 50% at end-of-induction in large multicenter trials [1–3], the potential target population of patients with refractory disease in bone/BM but not soft tissue is sizeable.

The study population was well defined. In contrast, other reports pose limitations on characterizing the efficacy of anti-G_D2_ mAbs against primary refractory HR-NB for the following reasons: (1) patients were in CR, precluding documentation of clinical response; (2) patients were heterogeneous, with primary and secondary refractory disease and with ≥ 1 prior relapses; (3) lack of details of extent-of-disease at the start of mAb therapy; (4) no documentation of anti-drug antibodies (e.g., HACA) which can compromise efficacy; and (5) usage with isotretinoin. [3, 7, 8, 17, 22]

None of the potentially prognostic clinical factors significantly impacted response or outcome (Table 3). That finding may be attributable to small numbers which limits validity of statistical analyses, but may also be due to the fact that 23/32 (72%) patients had previously received 2nd-line therapy and 20/32 (62.5%) patients had extensive disease (CS ≥ 10), limiting the impact of other clinical factors. The excellent responses with lower than the RP2D might relate to the fact that even the lowest dosage of 4.8 mg/kg/cycle (or ~ 144 mg/m^2^/cycle) is higher than the standard 70–100 mg/m^2^/cycle with other anti-G_D2_ mAbs. [7–9]

Of 9 patients enrolled after treatment with 3F8 or dinutuximab, 5 become long-term event-free survivors. The numbers are small but the findings suggest that naxitamab + GM-CSF could be considered for patients with persistent disease after treatment with other anti-G_D2_ mAbs.

The 3F8 + GM-CSF experience with primary refractory HR-NB was previously reported [11]. Comparability of results, however, warrants caution, not only because those patients also received isotretinoin, but more importantly because of major differences in disease burden between the study populations: only 3/32 (9%) naxitamab patients had BM-positivity with negative MIBG scans versus 29/72 (40%) m3F8 patients, and CS ≥ 10 were respectively present in 20/32 (62%) naxitamab patients versus 5/72 (7%) 3F8 patients. Of note, while all 32 naxitamab + GM-CSF patients in this report were evaluable for response, 23/72 3F8 patients received radiotherapy to their single MIBG-positive site (and had no BM metastases), making them inevaluable for response. With both naxitamab and 3F8, responses were usually rapid, evident after only 2 cycles.

Despite the large differences in disease burden described above, the CR rate of 75% in the current study was similar to the 68% with 3F8 + GM-CSF [11]. It is worth noting that during the era of 3F8, patients with extensive osteomedullary disease were judged not to be good candidates for anti-G_D2_ immunotherapy.

Regarding durability of response, 2-year PFS was 53% for naxitamab + GM-CSF, similar to the 48% with m3F8 + GM-CSF, but the 5-year PFS rate of 38% with naxitamab + GM-CSF compares favorably with the 24% with m3F8 + GM-CSF [11]. The common post-protocol treatment with an anti-NB vaccine [30] limits the ability to assess the impact of naxitamab + GM-CSF on PFS/OS. The vaccine is available for HR-NB patients who have completed standard treatment, including immunotherapy with anti-G_D2_ mAbs, and are in CR. It is designed to induce antibodies against G_D2_ and G_D3_ that could potentially mediate ablation of residual NB.

Five-year OS was similar for the current study (64%) and the prior study (65%) [11]. These high long-term OS rates point to the curability of relapse of HR-NB [10]. The results support the role of immunotherapy in improving survival of patients with persistent disease post-induction though the optimal immunotherapy regimen remains unknown. An encouraging salvage rate was observed among patients with isolated CNS relapse and relapse limited to a single bony site.

Radiotherapy to the primary site to prevent relapse is standard for HR-NB. Three patients received this pre-enrollment and 25 patients post-enrollment, including 16 after 1 cycle of naxitamab + GM-CSF. In addition, 12 patients received radiotherapy to 1–2 distant MIBG-avid skeletal sites. This did not preclude being assessable for response, given disease elsewhere. Overall, the radiotherapy could contribute to the durability of response.

Radiotherapy after initiation of immunotherapy is our long-standing practice, usually after 1 cycle with anti-G_D2_ mAb, a timing different from that in COG [21] and SIOPEN [22] where radiotherapy is completed before immunotherapy starts. Our rationale is to deliver immunotherapy as soon as possible, given predominant concern about metastatic disease. Further support for this timing might come from recent reports on preclinical and clinical studies that point to a possible vaccination effect when radiation is associated with immunotherapy. [32–34]

A wide variety of treatments have been used for HR-NB patients who have end-of-induction residual disease [3]. A common goal is to improve response enough to justify proceeding to consolidation with MAT. After receiving naxitamab, none of the patients in the current study underwent MAT even though MAT is standard-of-care elsewhere. The rationale for no MAT included several considerations. First, the study patients had chemo-resistant NB, yet MAT consists of potentially severely toxic chemotherapy [35, 36]. Second, MAT was prognostically favorable in randomized trials performed decades ago comparing MAT to no MAT [37–39], although long-term follow-up of one showed no survival advantage [40]. Those three trials have drawbacks undermining their current relevance, including suboptimal induction, different cytoreductive regimens, no routine radiotherapy, and no immunotherapy with anti-G_D2_ mAbs. Finally, MAT did not confer a survival advantage in the MSK experience with HR-NB involving retrospective analyses of patients treated non-randomly [9, 41, 42]. A randomized trial would be needed to reveal if MAT can be avoided.

Chemoimmunotherapy with co-administration of dinutuximab, irinotecan-temozolomide, and GM-CSF has recently gained widespread usage for chemo-resistant HR-NB due to highly encouraging results [29]. Chemoimmunotherapy using other anti-G_D2_ mAbs plus irinotecan-temozolomide or other chemotherapy has also shown promise [43–47]. Comparisons to identify which treatment—mAb + GM-CSF or chemoimmunotherapy—might be preferable specifically for osteomedullary disease are hindered by differences in the study populations, as well as details about the extent-of-disease. A randomized trial could answer the question. We co-administer naxitamab with chemotherapy for refractory HR-NB that includes soft tissue sites, given the lack of efficacy of anti-G_D2_ mAbs for NB in soft tissue. [5, 6]

In conclusion, naxitamb + GM-CSF is an attractive therapeutic option for patients with widespread chemo-resistant osteomedullary HR-NB and no prior PD, including post-induction. More experience will clarify if it is promising for patients previously treated with anti-G_D2_ mAbs. Outpatient treatment and low immunogenicity are additional welcome features of this immunotherapy.

## Data Availability

The data that support the findings of this study are available on request from the corresponding author. The data are not publicly available due to privacy or ethical restrictions.
